# ADAM12-L is a direct target of the miR-29 and miR-200 families in breast cancer

**DOI:** 10.1186/s12885-015-1108-1

**Published:** 2015-03-04

**Authors:** Sara Duhachek-Muggy, Anna Zolkiewska

**Affiliations:** Department of Biochemistry and Molecular Biophysics, Kansas State University, 141 Chalmers Hall, Manhattan, KS 66506 USA

**Keywords:** Metalloproteinase, Disintegrin, Alternative splicing, microRNA, Post-transcriptional gene regulation, Breast cancer, Claudin-low tumors, Epithelial-to-mesenchymal transition

## Abstract

**Background:**

*ADAM12-L* and *ADAM12-S* represent two major splice variants of human metalloproteinase-disintegrin 12 mRNA, which differ in their 3′-untranslated regions (3′UTRs). *ADAM12-L*, but not *ADAM12-S*, has prognostic and chemopredictive values in breast cancer. Expression levels of the two *ADAM12* splice variants in clinical samples are highly discordant, suggesting post-transcriptional regulation of the *ADAM12* gene. The miR-29, miR-30, and miR-200 families have potential target sites in the *ADAM12-L* 3′UTR and they may negatively regulate *ADAM12-L* expression.

**Methods:**

miR-29b/c, miR-30b/d, miR-200b/c, or control miRNA mimics were transfected into SUM159PT, BT549, SUM1315MO2, or Hs578T breast cancer cells. *ADAM12-L* and *ADAM12-S* mRNA levels were measured by qRT-PCR, and ADAM12-L protein was detected by Western blotting. Direct targeting of the *ADAM12-L* 3′UTR by miRNAs was tested using an *ADAM12-L* 3′UTR luciferase reporter. The rate of *ADAM12-L* translation was evaluated by metabolic labeling of cells with ^35^S cysteine/methionine. The roles of endogenous miR-29b and miR-200c were tested by transfecting cells with miRNA hairpin inhibitors.

**Results:**

Transfection of miR-29b/c mimics strongly decreased *ADAM12-L* mRNA levels in SUM159PT and BT549 cells, whereas *ADAM12-S* levels were not changed. *ADAM12-L*, but not *ADAM12-S*, levels were also significantly diminished by miR-200b/c in SUM1315MO2 cells. In Hs578T cells, miR-200b/c mimics impeded translation of *ADAM12-L* mRNA. Importantly, both miR-29b/c and miR-200b/c strongly decreased steady state levels of ADAM12-L protein in all breast cancer cell lines tested. miR-29b/c and miR-200b/c also significantly decreased the activity of an *ADAM12-L* 3′UTR reporter, and this effect was abolished when miR-29b/c and miR-200b/c target sequences were mutated. In contrast, miR-30b/d did not elicit consistent and significant effects on ADAM12-L expression. Analysis of a publicly available gene expression dataset for 100 breast tumors revealed a statistically significant negative correlation between *ADAM12-L* and both miR-29b and miR-200c. Inhibition of endogenous miR-29b and miR-200c in SUM149PT and SUM102PT cells led to increased *ADAM12-L* expression.

**Conclusions:**

The *ADAM12-L* 3′UTR is a direct target of miR-29 and miR-200 family members. Since the miR-29 and miR-200 families play important roles in breast cancer progression, these results may help explain the different prognostic and chemopredictive values of *ADAM12-L* and *ADAM12-S* in breast cancer.

## Background

Deregulated expression and activity of ADAM12 (A Disintegrin And Metalloproteinase 12) have been frequently observed in human breast cancer [1,2]. Overexpression of ADAM12 in the Polyoma virus middle T antigen (PyMT) mouse model of breast cancer accelerates tumor progression, and ADAM12 deficiency delays PyMT-induced mammary tumorigenesis [3,4]. The human *ADAM12* gene is the most frequently somatically mutated *ADAM* in breast cancer, and four missense mutations, D301H, G479E, T596A, and G668A, have a significant impact on protein functionality in cancer cells [5-7].

Human *ADAM12* mRNA is alternatively spliced, with several different transcript variants giving rise to distinct ADAM12 protein isoforms. Transcript variant 1 (exons 1-18 and 20-24, ~ 8,000 nt, RefSeq NM_003474) encodes a long, transmembrane protein isoform ADAM12-L. Transcript variant 2 (exons 1-19, ~3,400 nt, RefSeq NM_021641) gives rise to a short, secreted protein isoform ADAM12-S [8]. *ADAM12-L* and *ADAM12-S* mRNAs contain entirely different 3′ untranslated regions (3′UTRs) and are readily distinguishable by variant-specific probe-sets in several microarray platforms. Each of these two variants can further exist as an “a” or “b” form, which differ by a 9-nt extension at the end of exon 4. The “a” and “b” variants are not distinguishable in microarray profiling experiments [9].

There is a striking difference in the prognostic value of *ADAM12-L* and *ADAM12-S,* and the expression levels of these two *ADAM12* splice variants in clinical samples are highly discordant. *ADAM12-L*, but not *ADAM12-S*, is significantly elevated in the claudin-low molecular subtype of breast cancer, which has features of epithelial-mesenchymal transition (EMT), high expression of immune and endothelial genes, and gene expression signature reminiscent of mammary stem cells [10-13]. *ADAM12-L* is also induced during EMT in mammary epithelial cells [12,14-17], is enriched in mammary epithelial cells or breast cancer cells grown in suspension as mammospheres [12,18,19], is up-regulated in residual tumors remaining after endocrine therapy for estrogen receptor (ER)-positive disease [12,19,20], and the level of *ADAM12-L* expression predicts resistance to chemotherapy in ER-negative breast tumors [12,21-23]. In patients with lymph node-negative breast tumors who did not receive systemic treatment, *ADAM12-L* expression level is significantly associated with decreased distant metastasis-free survival times [24-27]. In contrast, *ADAM12-S* is not related to any of these characteristics [12,27].

The discrepancy between expression patterns of *ADAM12-L* and *ADAM12-S* in breast cancer clinical samples suggests that *ADAM12-L* expression may be regulated at the post-transcriptional level, through microRNAs targeting the unique 3′UTR present in this variant. Of particular interest are the miR-200, miR-29, and miR-30 families, which all have been linked to the mesenchymal phenotype, invasion, or metastasis in breast cancer [28,29], and which all have predicted target sites in the *ADAM12-L* 3′UTR, but not in the *ADAM12-S* 3′UTR. The miR-200 family, by forming a double-negative feedback loop with transcription factors of ZEB1 and ZEB2, is a key negative regulator of EMT and is down-regulated in breast cancer stem-like cells and in normal mammary stem/progenitor cells [29-33]. The miR-29 family, in particular miR-29b, is enriched in luminal breast cancers and inhibits metastasis by repressing regulators of angiogenesis, collagen remodeling, and tumor microenvironment [34]. Loss of miR-29b promotes a mesenchymal phenotype and increases metastasis. Furthermore, the miR-29 family members directly target Krüppel-like factor 4 (KLF4), a transcription factor required for the maintenance of breast cancer stem cells, and down-regulation of miR-29 family members results in increased stem-like properties *in vitro* and *in vivo* [35]. The miR-30 family appears to modulate the stem-like properties of breast cancer cells as well. Reduction of miR-30 levels was reported to promote self-renewal and to inhibit apoptosis in breast tumor-initiating cells [36]. Down-regulation of miR-30 family members was observed in non-adherent mammospheres compared to breast cancer cells under adherent conditions [37].

In this report, we asked whether ADAM12-L expression in breast cancer cells is regulated by members of the miR-200, miR-29, and miR-30 families. We established that transfection of miR-29b/c and miR-200b/c mimics strongly decreased the level of ADAM12-L protein in claudin-low SUM159PT, BT549, SUM1315MO2, and Hs578T cells, while miR-30b/d mimics had a more modest effect. Down-regulation of *ADAM12-L* by miR-29b/c and miR-200b/c occurred at the post-transcriptional level and was mediated through direct targeting of the *ADAM12-L* 3′UTR, resulting in either target mRNA degradation or decreased translation, depending upon the cell line studied. Importantly, we found a significant negative correlation between *ADAM12-L* and both miR-29b and miR-200c in breast invasive carcinomas. Inhibition of the endogenous miR-29b and miR-200c with miRNA hairpin inhibitors increased the level of *ADAM12-L* mRNA in SUM149PT and SUM102PT cell lines. These results underscore a novel post-transcriptional mode of regulation of ADAM12 expression and help explain the different prognostic and chemopredictive value of *ADAM12-L* and *ADAM12-S* in breast cancer.

## Methods

### Approvals

The Institutional Biosafety Committee at Kansas State University approved all experiments performed in this project (IBC Protocol #942). We did not perform any human or animal studies. Our analysis of human data resulted from mining previously published datasets.

### Reagents

MiRIDIAN microRNA mimics, mimic negative control, microRNA hairpin inhibitors, and hairpin inhibitor negative control were obtained from Dharmacon. The *ADAM12-L* 3′UTR luciferase reporter construct containing nt 3097-6065 from the *ADAM12-L* transcript was obtained from Origene. Anti-ADAM12-L rabbit polyclonal antibody (#3394), raised against the cytoplasmic domain of human ADAM12-L, was generated in our laboratory, as previously described [27]. This antibody was used for immunoblotting at a 1:10,000 dilution, with overnight incubation. Anti-α-tubulin mouse monoclonal antibody was obtained from Sigma (clone DM1A) and used at a 1:200,000 dilution.

### Cell culture

SUM149PT, SUM159PT, and SUM1315MO2 cell lines were obtained from Asterand (Detroit, MI). BT549 and Hs578T cells were obtained from American Type Culture Collection (Manassas, VA). SUM102PT cells were a gift from Dr. Fariba Behbod (University of Kansas Medical Center). SUM149PT and SUM159PT cells were cultured in Ham’s F-12 medium supplemented with 5% fetal bovine serum (FBS), 10 mM HEPES, 5 μg/ml insulin, and 1 μg/ml hydrocortisone. SUM1315MO2 cells were cultured in Ham’s F-12 medium supplemented with 5% FBS, 10 mM HEPES, 10 ng/ml epidermal growth factor, and 5 μg/ml insulin. BT549 cells were cultured in RPMI-1640 medium supplemented with 10% FBS, 1 mM pyruvate, and 0.8 μg/ml insulin. Hs578T cells were cultured in Dulbecco’s Modified Eagle Medium (DMEM) supplemented with 10% FBS and 10 μg/ml insulin. SUM102PT were culture in Ham’s F-12 medium supplemented with 5% FBS, 1 μg/ml hydrocortisone, 5 μg/ml insulin and 1% penicillin/streptomycin/Fungizone. Cells were maintained at 37°C under humidified atmosphere containing 5% CO_2_.

### Cell transfections

Cells were seeded onto new plates one day prior to transfection. MicroRNA mimics and hairpin inhibitors were resuspended in 1× siRNA buffer (Dharmacon) and transfected at a final concentration of 50 nM and 100 nM, respectively, using DharmaFECT 1 transfection reagent (Dharmacon). Transfection complexes were removed after 24 hours, and cells were analyzed 48-72 hours later. Plasmid transfection was performed using X-tremeGENE HP transfection reagent (Roche) and 0.1 μg DNA per well in 24-well plates, at a 2:1 reagent:DNA ratio. For cells transfected with both miRNA and plasmid DNA, the transfections were performed sequentially, with the miRNA mimics introduced first and the plasmid introduced the following day. Targeted down-regulation of ZEB1 by miR-200b/c was used as positive control. The transfection conditions used throughout the paper to target ADAM12-L caused ZEB1 protein knock-down to undetectable levels by miR-200b/c mimics in SUM159PT, SUM1315MO2, and Hs578T cells, and decreased the *ZEB1* 3′UTR reporter in SUM159PT cells by 50%.

### Western blotting

Cells were treated with lysis buffer (50 mM Tris-HCl pH 7.4, 150 mM NaCl, 1% Triton X-100, 0.5% sodium deoxycholate, 0.1% sodium dodecylsulfate, 5 mM EDTA, 1 mM 4-(2-Aminoethyl) benzenesulfonyl fluoride hydrochloride (AEBSF), 5 μg/ml pepstatin, 5 μg/ml leupeptin, 5 μg/ml aprotinin, and 10 mM 1,10-phenanthroline). Extracts were centrifuged for 15 minutes at 16,000g at 4°C. After centrifugation, the supernatants were directly analyzed by Western blotting using anti-tubulin antibody or incubated with concanavalin A agarose (Sigma; 50 μl resin per 1 ml cell lysate) for 2 hours at 4°C to enrich for glycoproteins. The resin was washed three times and the glycoproteins were eluted with 3× SDS gel loading buffer. Proteins were resolved using SDS-PAGE (8% gel) and were transferred to a nitrocellulose membrane. The membrane was stained with Ponceau S and an image was saved. The membrane was blocked using 5% milk and 0.3% Tween-20 in Dulbecco’s Phosphate Buffered Saline (DPBS). Primary antibody was diluted in blocking buffer and incubated with the membrane. Horseradish peroxidase-conjugated anti-rabbit or anti-mouse antibody was used as a secondary antibody. Detection was performed using the SuperSignal West Pico Chemiluminescent Substrate (Pierce). Each experiment was repeated independently at least two times; representative blot images are shown.

### 3′UTR luciferase reporter assays

Cells were sequentially transfected with miRNA mimics and the 3′UTR reporter plasmids, as described above. A *Renilla* luciferase vector, pRL-TK (Promega) was co-transfected with the reporter plasmid as a transfection control. Forty eight hours after vector transfection, the cells were washed with DPBS containing calcium and magnesium and then lysed using 1× Passive Lysis Buffer (Promega), according to the manufacturer’s instructions. The lysates were analyzed for firefly and *Renilla* luciferase activities using the Dual Luciferase Reporter Assay System (Promega).

### Mutagenesis

The predicted miR-29, miR-30, and two miR-200 target sites in the *ADAM12-L* 3′UTR reporter plasmid were mutated by site-directed mutagenesis. The primers to mutate the miR-29 site were: 5′-TGC TGT GCT GTG CTA *CTT* TGC TCT GTC TAC TTG C-3′ (F) and the reverse complement. The primers to mutate the miR-30 site were: 5′- TAT ACT ATT AAA AAG *TCC* TAC AGA ATT TTA TGG-3′ (F) and the reverse complement. The primers used to mutate the first miR-200 site were: 5′-TTC CCT TAC AAT ATG GA*T CT*T ATT AAT CCT TCC AAG A-3′ (F) and the reverse complement. The primers used to mutate the second miR-200 site were: 5′-TTA ATC CTT CCA AGA TG*T CT*T ATT TAT CAA GTG AAG C-3′ (F) and the reverse complement. The italicized portions represent the mutated bases. The presence of mutations was confirmed by DNA sequencing.

### ^35^S metabolic labeling of cells

Hs578T cells were transfected with microRNA mimics or mimic control, as described above. Two days after transfection, cells were washed and incubated in labeling media (9 parts DMEM without cysteine and methione: 1 part complete DMEM), containing 80 μCi/ml EasyTag EXPRESS^35^S Protein labeling mix (PerkinElmer). After labeling for the indicated times, cell lysates were prepared and ADAM12-L was immunoprecipitated using antibody #3394 and Protein G Sepharose. Pre-immune serum was used as a control. The immunocomplexes were analyzed by SDS-PAGE and autoradiography. The experiment was repeated independently two times.

### cDNA preparation and qRT-PCR analysis

Total RNA was extracted using the Qiagen RNeasy kit and was subjected to on-column digestion with deoxyribonuclease I (Qiagen). One microgram of the total RNA was reverse-transcribed using the SuperScript III First Strand Synthesis System (Life Technologies) and oligo(dT) primers. Real time quantitative PCR (qRT-PCR) was performed using 15 μl volumes in a 96-well format on a CFX96 cycler. The final reaction mixture contained 7.5 μl iQ SYBRgreen Supermix (BioRad), 6 μl diluted cDNA (1:10 for *ADAM12* analysis and 1:100 for *ACTIN* analysis) and 0.5 μM primers. The primers used for *ADAM12-L* analysis were 5′-AGC CAC ACC AGG ATA GAG AC-3′ (F) and 5′-CGC CTT GAG TGA CAC TAC AG-3′ (R). The primers used for the *ADAM12-S* analysis were 5′-TCC ATC CAA GCA AAC TGA AT-3′ (F) and 5′-GTT GGT GAC TCT GTG GGT TC-3′ (R). The primers used for *ACTIN* analysis were 5′-TTG CCG ACA GGA TGC AGA A-3′ (F) and 5′-GCC GAT CCA CAC GGA GTA CT-3′ (R). The PCR conditions were: 95°C, 10 s; 60°C, 15 s; 72°C, 30 s. At the conclusion of each run, a melt curve analysis was performed to ensure that a single product had been synthesized. The relative expression of *ADAM12*, normalized to *ACTIN*, was calculated using the 2^-ΔΔCt^ method.

### Data mining

*ADAM12-L* and *ADAM12-S* expression data for a panel of breast cancer cell lines were retrieved from Gene Expression Omnibus (GEO) (http://www.ncbi.nlm.nih.gov/geo/) and ArrayExpress (http://www.ebi.ac.uk/arrayexpress/). The microRNA expression data for a panel of breast cancer cell lines were obtained from the online supplemental material from Riaz et al. [38]. *ADAM12-L* and miRNA expression data for a cohort of 100 human breast tumors were retrieved from GEO. Expression values were log_2_-transformed and median-centered.

### Statistics

Correlation and *t* test analyses were performed using the GraphPad Prism 6.0 software.

## Results

**Figure 1:**
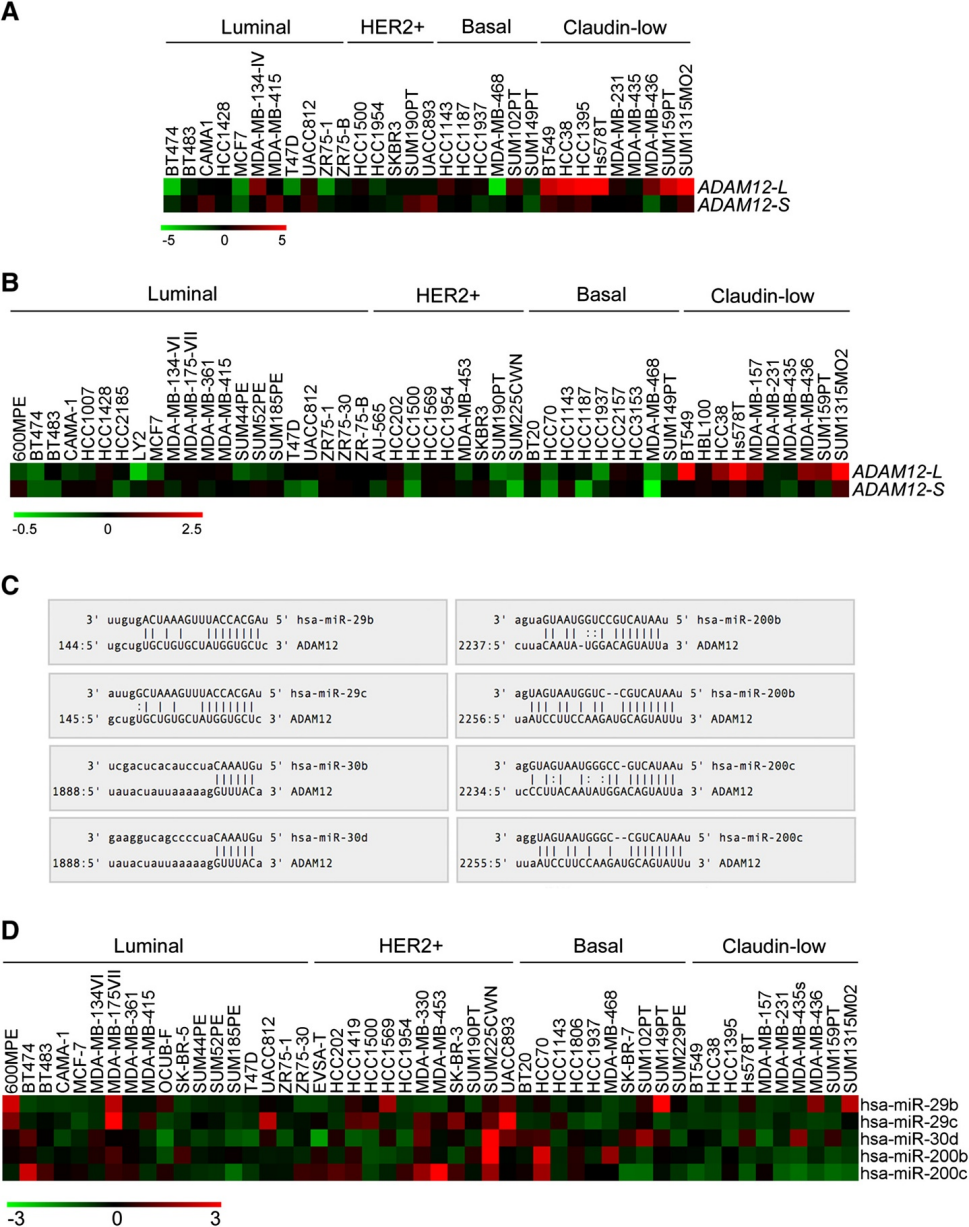
**Pattern of expression of*ADAM12-L*,*ADAM12-S*, and miRNAs in breast cancer cell lines. (A)** Discrepancy between *ADAM12-L* and *ADAM12-S* levels in a panel of human breast cancer cells profiled with the Agilent 4×44K UNC custom microarray platform, based on ref. [39]. The expression data were retrieved from GEO:GSE50470. Expression values of *ADAM12-L* were calculated as the average readouts for three probes (A_23_P202327, NM_003474_2_4965, and NM_003474_2_4854). Expression values of *ADAM12-S* are based on the A_23_P350512 probe. **(B)** Discrepancy between *ADAM12-L* and *ADAM12-S* expression levels in a panel of human breast cancer cells profiled with the Affymetrix HG-U133A platform, based on ref. [40]. The expression data were retrieved from ArrayExpress, accession number E-TABM-157. **(C)** Predicted miR-29b/c, miR-30b/d, and miR-200b/c target sites in the human *ADAM12-L* 3′UTR, based on TargetScan Release 6.2. **(D)** miR-29b, miR-29c, miR-30d, miR-200b, and miR-200c levels in a panel of breast cancer cell lines, based on ref. [38]. Expression data for miR-30b were not available. Downloaded data were log_2_-transformed, median-centered and Z scores were calculated. In **A**, **B**, and **D**, each colored square in the heatmaps represents the relative transcript abundance, in log_2_ space. Expression values were median-centered across all cell lines.

Our previous analysis of a number of gene expression profiles of human breast cancers revealed significant discrepancies between *ADAM12-L* and *ADAM12-S* expression levels [12]. Here, we examined *ADAM12-L* and *ADAM12-S* levels in a panel of breast cancer cell lines, which were previously profiled using two different microarray platforms: an Agilent 4×44K platform (ref. [39], Figure 1A) or an Affymetrix HG-U133A platform (ref. [40], Figure 1B). In both cases, *ADAM12-L* was strongly up-regulated in claudin-low cell lines, whereas the level of *ADAM12-S* in claudin-low cells did not significantly differ from the rest of the cell lines. This expression pattern of *ADAM12-L* and *ADAM12-S* in cell lines mirrored their expression patterns in clinical tumor samples [12].

Selective up-regulation of *ADAM12-L* in claudin-low samples raised the possibility that *ADAM12-L* expression might be repressed by one or more miRNAs, which are down-regulated in claudin-low tumors/cell lines and which could directly target the sites present in the *ADAM12-L* 3′UTR. We focused on the miR-29, miR-30, and miR-200 families, which act as tumor suppressors in breast cancer. The miR-29 family consists of three members with the same seed sequence, miR-29a-c. The miR-30 family is made up of 5 members, miR-30a-e. The miR-200 family consists of five members: miR-200a-c, miR-141 and miR-429. We have selected to study two representative miRNAs from each family: miR-29b (a potent inhibitor of breast tumor metastasis [34]) and miR-29c (associated with a significantly reduced risk of dying from breast cancer [41]), miR-30b and miR-30d (both significantly down-regulated in ER-negative and progesterone receptor (PR)-negative breast tumors [42]), and miR-200b and miR-200c (both representing key negative regulators of EMT and anoikis resistance [30-32]). The 3′UTR of human *ADAM12-L* contains well conserved potential target sites for miR-29b/c, miR-30b/d, and two poorly conserved potential sites for miR-200b/c (Figure 1C). miRNA profiling of 51 breast cancer cell lines has previously established that miR-29b/c, miR-30d, and miR-200b/c are under-expressed in claudin-low breast cancer cell lines (ref. [38], Figure 1D; miR-30b was not measured in the referenced study).

**Figure 2:**
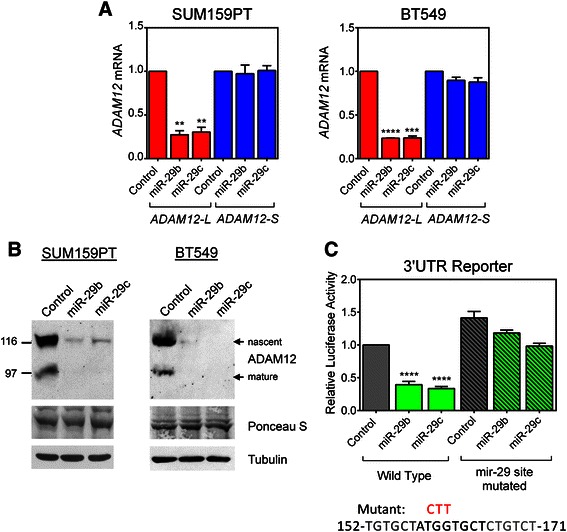
***ADAM12-L*, but not*ADAM12-S*, is a target for miR-29b/c. (A**,**B)** SUM159PT and BT549 cells were transfected with miR-29b mimic, miR-29c mimic, or mimic control. **(A)***ADAM12-L* and *ADAM12-S* mRNA levels were measured by qRT-PCR and normalized to *β-ACTIN*. Fold changes in miRNA-transfected cells *versus* control cells were calculated. Graphs represent average values obtained in three independent experiments ± SEM. Statistical significance was determined by one-sample *t* tests. **P < 0.01, ***P < 0.001, ****P < 0.0001. **(B)** Cell lysates were enriched for glycoproteins and analyzed by Western blotting using an anti-ADAM12-L antibody. The nascent, full-length form and the mature, processed form are indicated. A Ponceau S-stained band in the glycoprotein-enriched fraction and tubulin in total cell lysates were used as loading controls. **(C)***Upper* SUM159PT cells were transfected with miR-29b, miR-29c mimics, or mimic control and then with the indicated *ADAM12-L* 3′UTR reporter or an empty vector and a *Renilla* luciferase control vector. The firefly luciferase activity was measured after 48 h and was normalized to *Renilla* luciferase activity and to the empty vector. Graph shows the average values for at least two independent experiments ± SEM. Significance was determined by one-sample *t* tests. ****P < 0.0001. *Lower* Three nucleotides in the putative miRNA target site (shown in bold) were mutated to destroy the site. The mutated residues are shown in red above the wild-type sequence. The position in the *ADAM12-L* 3′UTR relative to the stop codon is indicated.

To determine whether low levels of miR-29b/c are required for high expression of *ADAM12-L* in claudin-low cell lines, we utilized SUM159PT and BT549 cells, two representative claudin-low cell lines with low endogenous levels of miR-29b/c (see Figure 1D). Cells were transfected with miR-29b/c or control miRNA mimics, and the levels of *ADAM12-L* and *ADAM12-S* mRNAs were measured three days later by qRT-PCR. We found that miR-29b/c mimics decreased the level of *ADAM12-L* by ~70%, and that this effect was statistically significant (Figure 2A). *ADAM12-S* expression was not significantly altered by transfection with miR-29b/c mimics. In parallel experiments, we examined the effects of miR-29b/c on ADAM12-L protein expression by immunoblotting. We observed that both miR-29b and miR-29c strongly diminished the level of ADAM12-L protein in both cell lines (Figure 2B). Testing the effect of miRNAs on the expression level of the ADAM12-S isoform was not possible because specific antibodies against ADAM12-S are not currently available. Decreased ADAM12-L protein and mRNA levels after transfection of miR-29b/c suggested that these miRNAs might be directly targeting the *ADAM12-L* 3′UTR. To examine this possibility, we performed a miRNA target reporter luciferase assay using the pMirTarget reporter vector comprising a ~3-kb region of the *ADAM12-L* 3′UTR down-stream of the firefly luciferase gene. An approximately 50-60% reduction in the luciferase activity was observed in miR-29b/c mimic-transfected SUM159PT cells compared to control mimic-transfected cells (Figure 2C). Disruption of the predicted miR-29 target site by site-directed mutagenesis largely diminished the effects of miR-29b/c.

**Figure 3:**
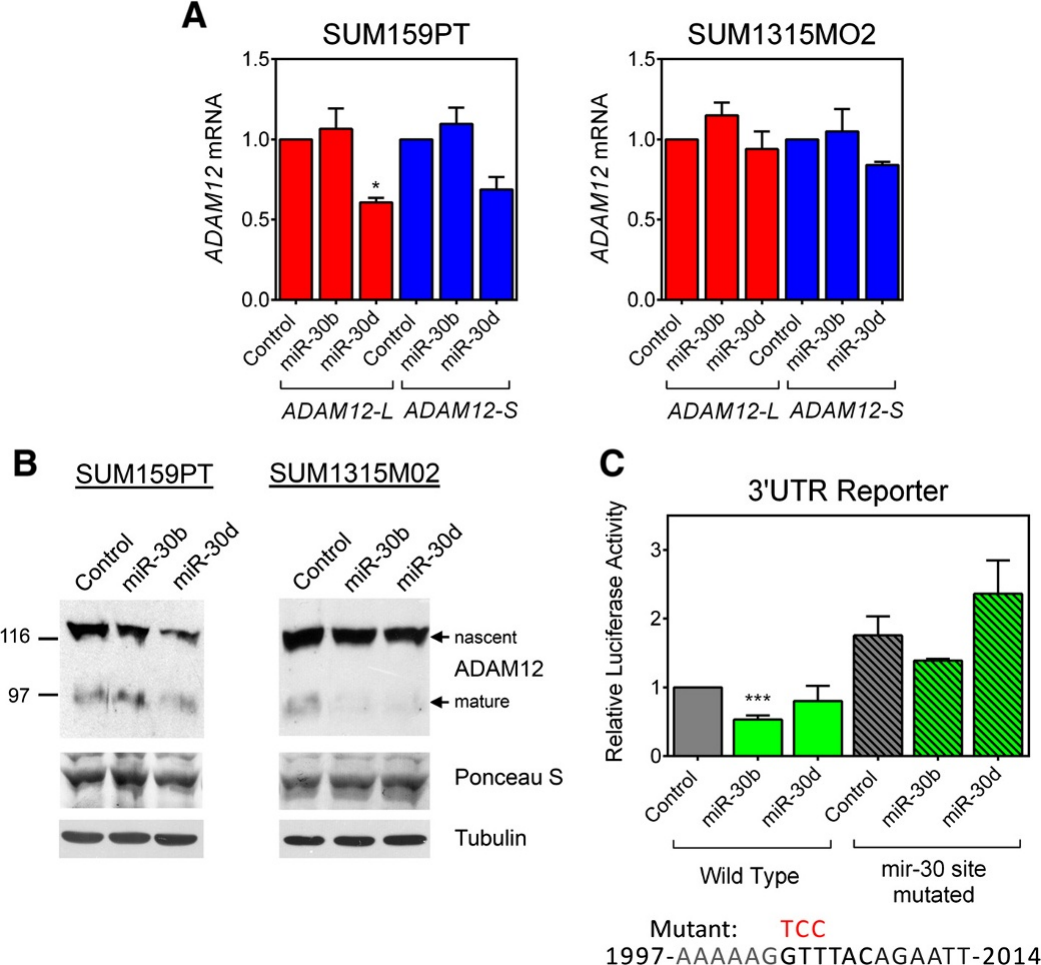
***ADAM12-L*is a poor target for miR-30b/d. (A,B)** SUM159PT and SUM1315MO2 cells were transfected with miR-30b mimic, miR-30d mimic, or mimic control. **(A)***ADAM12-L* and *ADAM12-S* mRNA levels were measured by qRT-PCR and normalized to *β-ACTIN*. Fold changes in miRNA-transfected cells *versus* control cells were calculated. Graphs represent average values obtained in three (for SUM159PT) or two (for SUM1315MO2) independent experiments ± SEM. Statistical significance was determined by one-sample *t* tests. *P < 0.05. **(B)** Cell lysates were enriched for glycoproteins and analyzed by Western blotting using an anti-ADAM12-L antibody. The nascent, full-length form and the mature, processed form are indicated. A Ponceau S-stained band in the glycoprotein-enriched fraction and tubulin in total cell lysates were used as loading controls. **(C)***Upper* SUM159PT cells were transfected with miR-30b, miR-30d mimics, or mimic control and then with the indicated *ADAM12-L* 3′UTR reporter or an empty vector and a *Renilla* luciferase control vector. The firefly luciferase activity was measured after 48 h and was normalized to *Renilla* luciferase activity and to the empty vector. Graph shows the average values for at least two independent experiments ± SEM. Significance was determined by one-sample *t* tests. ***P < 0.001. *Lower* Three nucleotides in the putative miRNA target site (shown in bold) were mutated to destroy the site. The mutated residues are shown in red above the wild-type sequence. The position in the *ADAM12-L* 3′UTR relative to the stop codon is indicated.

Similarly, we assessed whether miR-30b/d potentially target *ADAM12-L*. We transfected miR-30b/d or control mimic into SUM159PT and SUM1315MO2 cells, two claudin-low cell lines with low to moderate endogenous miR-30b/d expression (Figure 1D), and measured the level of *ADAM12-L* and *ADAM12-S* mRNA by qRT-PCR. miR-30d exerted a ~30%, statistically significant, down-regulation of *ADAM12-L* expression in SUM159PT cells and no apparent inhibition of *ADAM12-L* expression in SUM1315MO2 cells. miR-30b did not diminish *ADAM12-L* levels in either cell line and neither miRNA mimic affected *ADAM12-S* expression (Figure 3A). miR-30b/d had a modest effect on ADAM12-L protein in both cell lines (Figure 3B). To test whether miR-30b or miR-30d directly targets the *ADAM12-L* 3′UTR, we used the luciferase reporter in SUM159PT cells. Transfection of miR-30b mimic elicited a significant decrease in luciferase activity but miR-30d mimic did not (Figure 3C). Destruction of the potential miR-30 target site by mutagenesis eliminated the effect of miR-30b mimic.

**Figure 4:**
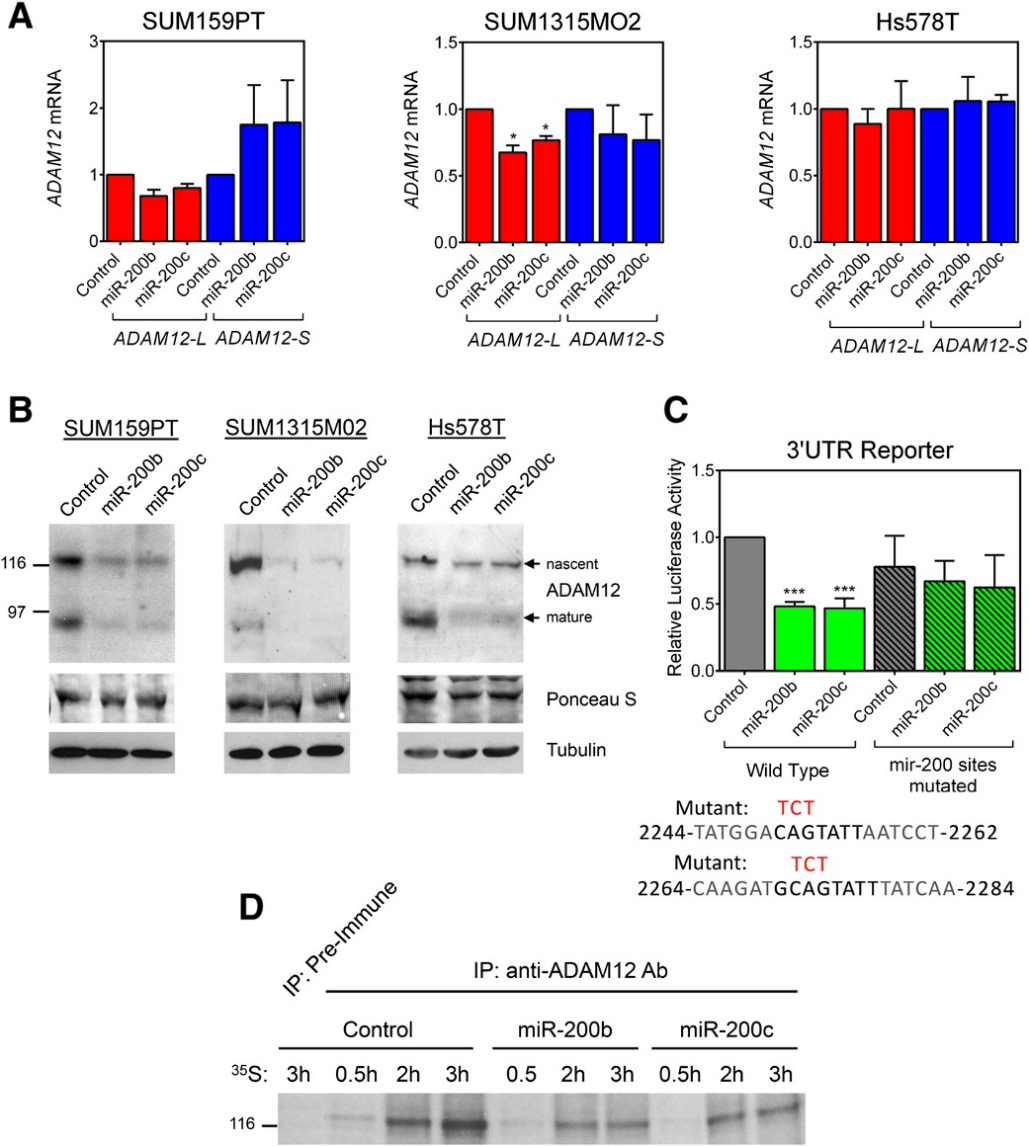
***ADAM12-L*, but not*ADAM12-S*, is a target for miR-200b/c. (A**,**B)** SUM159PT, SUM1315MO2 and Hs578T cells were transfected with miR-200b mimic, miR-200c mimic, or mimic control. **(A)***ADAM12-L* and *ADAM12-S* mRNA levels were measured by qRT-PCR and normalized to *β-ACTIN*. Fold changes in miRNA-transfected cells *versus* control cells were calculated. Graphs represent average values obtained in three independent experiments ± SEM. Statistical significance was determined by one-sample *t* tests. *P < 0.05. **(B)** Cell lysates were enriched for glycoproteins and analyzed by Western blotting using an anti-ADAM12-L antibody. The nascent, full-length form and the mature, processed form are indicated. A Ponceau S-stained band in the glycoprotein-enriched fraction and tubulin in total cell lysates were used as loading controls. **(C)***Upper* SUM159PT cells were transfected with miR-200b, miR-200c mimics, or mimic control and then with the indicated *ADAM12-L* 3′UTR reporter or an empty vector and a *Renilla* luciferase control vector. The firefly luciferase activity was measured after 48 h and was normalized to *Renilla* luciferase activity and to the empty vector. Graph shows the average values for at least three independent experiments ± SEM. Significance was determined by one-sample *t* tests. ***P < 0.001. *Lower* Three nucleotides in each putative miRNA target site (shown in bold) were mutated to destroy the site. The mutated residues are shown in red above the wild-type sequences. The positions in the *ADAM12-L* 3′UTR relative to the stop codon are indicated. **(D)** Hs578T cells were transfected with miR-200b mimic, miR-200c mimic, or mimic control. Forty-eight hours after transfection, cells were treated with ^35^S methionine/cysteine for the indicated periods of time, followed by immunoprecipitation with anti-ADAM12-L antibody or pre-immune serum, SDS-PAGE and autoradiography. The nascent, full-length form of ADAM12-L (~120 kDa) is shown.

To study the effects of miR-200b/c mimics, we selected SUM159PT, SUM1315MO2, and Hs578T cells, which all express low levels of endogenous miR-200b/c (Figure 1D). Transfecting miR-200b/c diminished *ADAM12-L* expression by ~20-30% in SUM159PT and SUM1315MO2 cells. This effect was statistically significant in SUM1315MO2 cells, but it did not reach statistical significance in SUM159PT cells (Figure 4A). Strikingly, Hs578T cells showed no change in *ADAM12-L* levels after transfection of miR-200b/c mimics. *ADAM12-S* levels were unchanged in all three cell lines (Figure 4A). Interestingly, ADAM12-L protein levels in SUM159PT, SUM1315MO2, or HS578T cells were strongly down-regulated after transfection of miR-200b/c mimics (Figure 4B), despite modest or negligible effects of these mimics on *ADAM12-L* mRNA levels. Targeting the 3′UTR of *ADAM12-L* by miR-200b/c was further assessed by luciferase reporter assays in SUM159PT cells. Both miR-200b and miR-200c mimics elicited a statistically significant, ~50% decrease in the luciferase activity, which was abolished when the two putative miR-200b/c target sites were destroyed (Figure 4C).

MiRNAs can reduce protein expression by inducing mRNA degradation or by reducing the rate of mRNA translation [43]. Since miR-200b/c mimics had no detectable effect on *ADAM12-L* mRNA level but they strongly reduced ADAM12-L protein in Hs578T cells, we asked whether miR-200b/c might have reduced the rate of *ADAM12-L* translation in Hs578T cells. Cells were transfected with miR-200b/c mimics (or control mimic) and, three days later, we performed metabolic cell labeling with ^35^S cysteine/methionine. After the indicated periods of time, the cell lysates were subjected to immunoprecipitation with an ADAM12-L antibody or pre-immune serum, followed by SDS-PAGE and autoradiography. We observed that the amount of ^35^S-labeled nascent form of ADAM12-L protein in miR-200b/c mimic-transfected cells was substantially lower than the amount of ^35^S-labeled ADAM12-L in control mimic-treated cells (Figure 4D). These results suggest that the main mechanism by which miR-200b/c reduced ADAM12-L expression in Hs578T cells was most likely through the inhibition of *ADAM12-L* mRNA translation.

**Figure 5:**
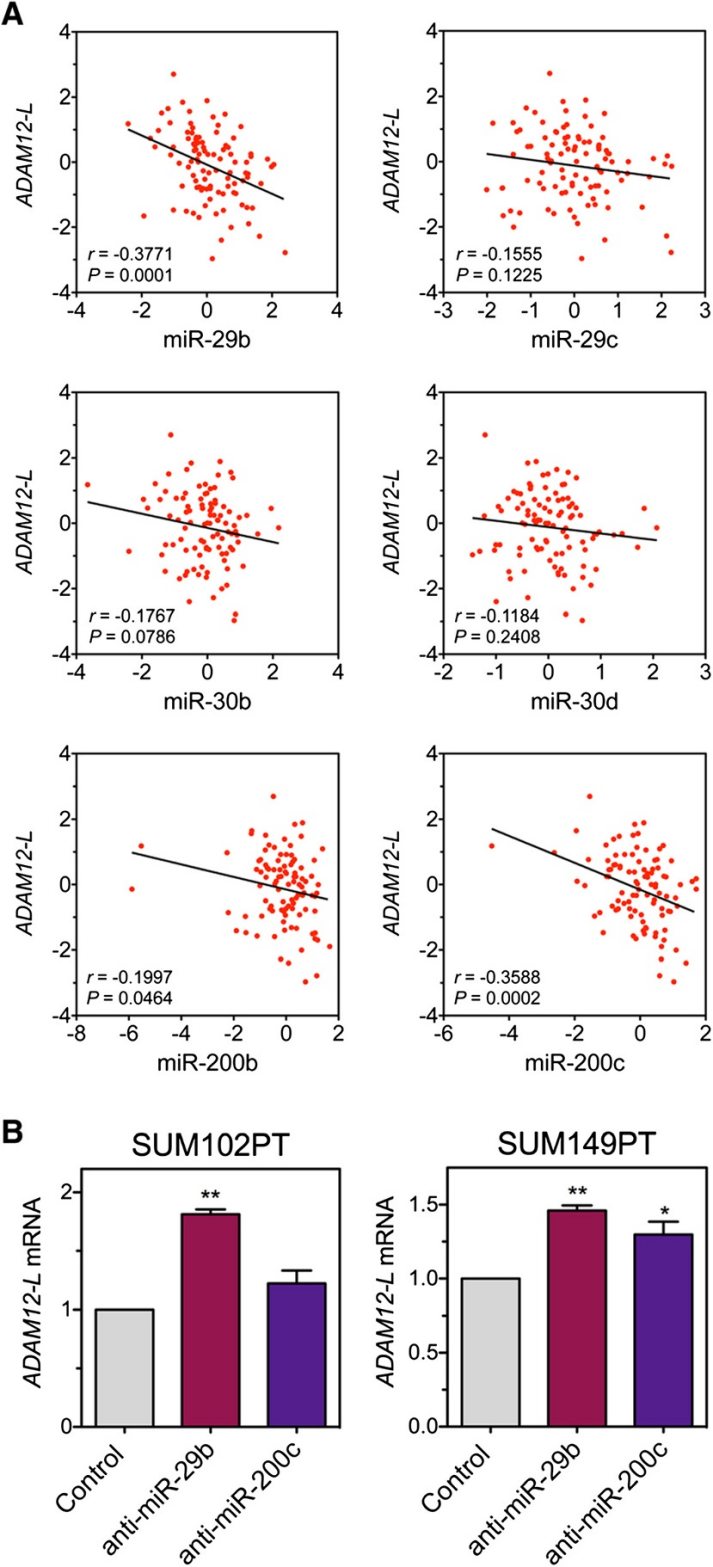
**Relationship between endogenous miR-29b, miR-200c, and*ADAM12-L*in breast tumors and breast cancer cell lines. (A)** Correlation between *ADAM12-L* and miRNA levels in a set of 100 human breast tumors profiled with the Agilent Whole Human Genome Microarray 4×44K G4112F and the Agilent Human miRNA Microarray 2.0 G4470B, based on ref. [44]. The expression data were retrieved from GEO:GSE19536. Expression values of *ADAM12-L* were based on the A_23_P202327 probe. The expression values were median-centered for all tumors. Pearson *r* and *P* values are shown for each comparison. **(B)** SUM102PT and SUM149PT cells were transfected with miRNA hairpin inhibitors targeting miR-29b or miR-200c, or with hairpin inhibitor control. *ADAM12-L* mRNA levels were measured by qRT-PCR and normalized to *β-ACTIN*. Fold changes in miRNA inhibitor-transfected cells *versus* control cells were calculated. Graphs represent average values obtained in three independent experiments ± SEM. Statistical significance determined by one-sample *t* tests. *P < 0.05, **P < 0.01.

To determine whether miR-29b/c, miR-30b/d, or miR-200b/c might regulate ADAM12-L expression in breast cancer patients *in vivo*, we examined the relationship between these miRNAs and *ADAM12-L* mRNA in a cohort of 100 breast cancer patients for which mRNA/miRNA expression data were publicly available (GEO: GSE19536) [44]. Importantly, the microarray platform used in the referenced study contained an oligoprobe mapping uniquely to the *ADAM12-L* transcript, without contribution of the *ADAM12-S* splice variant. There was a significant negative correlation between miR-29b and *ADAM12-L* (*P* = 0.0001), between miR-200c and *ADAM12-L* (*P* = 0.0002), and a weaker but significant correlation between miR-200b and *ADAM12-L* (*P* = 0.0464) (Figure 5A). These results are consistent with a role of miR-29b and miR-200c (and possibly miR-200b) in the regulation of *ADAM12-L* expression in breast tumors. To further test this hypothesis, we asked whether inhibition of the endogenous miR-29b or miR-200c in SUM102PT and SUM149PT, two basal cell lines with low to moderate expression of miR-29b and miR-200c (see Figure 1D), is sufficient to increase the level of *ADAM12-L*. We transfected these cells with miRNA hairpin inhibitors to miR-29b and miR-200c (or with control hairpin inhibitor) and assessed the level of *ADAM12-L* mRNA by qRT-PCR. In SUM102PT cells, miR-29b inhibitor increased the *ADAM12-L* level by ~80%, and this effect was significant. miR-200b/c inhibitor increased *ADAM12-L* by ~20%, but this effect did not reach the level of statistical significance (Figure 5B). In SUM149PT cells, miR-29b and miR-200c inhibitors increased *ADAM12-L* levels by ~50% and ~30%, respectively, and these effects were statistically significant (Figure 5B).

## Discussion

In this report, we examined whether three miRNA families, miR-29, miR-30, and miR-200, directly target the *ADAM12-L* 3′UTR in human breast cancer cells. Since the *ADAM12-S* 3′UTR lacks predicted target sites for these miRNA families and since miR-29, miR-30, or miR-200 levels are highly variable in breast cancer, selective targeting of the *ADAM12-L* 3′UTR by these miRNAs might explain why *ADAM12-L* and *ADAM12-S* expression patterns in breast tumors *in vivo* and in response to experimental manipulations *in vitro* often differ significantly.

Among the three miRNA families tested, miR-30 elicited the least consistent effects. While miR-30b diminished the *ADAM12-L* 3′UTR reporter activity, the level of *ADAM12-L* mRNA in SUM159PT and SUM1315MO2 cells was not affected upon transfection of miR-30b. In contrast, miR-30d seemed to down-regulate *ADAM12-L* in SUM159PT cells, but this effect was not reproduced in SUM1315MO2 cells, and the *ADAM12-L* 3′UTR reporter activity was not diminished in response to miR-30d. Both miR-30b and miR-30d had only minor effects on ADAM12-L protein levels in SUM159PT and SUM1315MO2 cells. We conclude that the miR-30 family does not contribute significantly to the regulation of ADAM12-L expression in the two cell lines examined here.

In contrast, miR-29b/c consistently produced strong down-regulation of ADAM12-L expression at the mRNA and protein levels in both SUM159PT and BT549 cell lines, and they decreased the *ADAM12-L* 3′UTR reporter activity in SUM159PT cells. Mutation of the single miR-29 target site in the *ADAM12-L* 3′UTR blunted the effect of miR-29b/c on the reporter activity, confirming direct targeting of the *ADAM12-L* 3′UTR region by miR-29b/c. The levels of the *ADAM12-S* splice variant were not changed by miR-29b/c, consistent with the lack of any predicted miR-29 target sites in the *ADAM12-S* 3′UTR.

The miR-29 family was reported previously to target the *Adam12* transcript in NIH3T3 cells [45]. miR-29 has been also implicated in the regulation of *Adam12* expression in response to transforming growth factor β (TGFβ) in experimental renal fibrosis in mice [46]. *Adam12* is the only splice variant known to exist in mice and, similar to human *ADAM12-L*, it contains a miR-29 target site. In humans, *ADAM12-L* was identified as one of the direct targets of miR-29b in trabecular meshwork cells, and increased expression of *ADAM12-L* in response to oxidative stress-induced down-regulation of miR-29b may contribute to the elevation of intra-ocular pressure in glaucoma [47]. In the context of breast cancer, miR-29b has been recently identified as a part of a GATA3-miR-29b axis, which regulates the tumor microenvironment and inhibits metastasis [34]. Down-regulation of miR-29 members also results in increased expression of the transcription factor KLF4 and expansion of stem-like cell populations *in vitro* and *in vivo* [35]. The miR-29 family is down-regulated in claudin-low cell lines and tumors, in which *ADAM12-L*, but not *ADAM12-S*, is strongly elevated. Thus, increased expression of *ADAM12-L* in claudin-low cell lines and tumors could be facilitated, at least in part, by low levels of miR-29 family members.

The third miRNA family tested here, miR-200, has not been previously reported to regulate ADAM12 expression. We have found that two members of this family, miR-200b and miR-200c, strongly diminished ADAM12-L protein in SUM159PT, SUM1315MO2, and Hs578T cells. The decrease in *ADAM12-L* mRNA was, however, more modest or, in the case of Hs578T cells, no change in *ADAM12-L* was detected in miR-200b/c-transfected cells. This apparent discrepancy between the effects of miR-200b/c on ADAM12-L protein and mRNA levels led us to investigate whether miR-200b/c might block translation of *ADAM12-L* mRNA, as miRNA are known to regulate mRNA stability and/or translation [43]. Indeed, we observed a slower rate of ADAM12-L protein synthesis in Hs578T cells treated with miR-200b/c mimics than in cells treated with control miRNA mimics. The question of why the inhibition of ADAM12-L by miR-200b/c expression in some cells (such as Hs578T) occurs at the translational level, and why in other cells (such as SUM159PT and SUM1315MO2) involves a decrease in steady-state *ADAM12-L* mRNA levels, remains open. Finally, the *ADAM12-L* 3′UTR reporter activity was significantly reduced by miR-200b/c, despite the fact that the two predicted miR-200 target sites present in the *ADAM12-L* 3′UTR are not well conserved between species. Mutations within these two miR-200 target sites abolished the effect of transfected miR-200b/c mimics, suggesting direct interaction between miR-200b/c and the *ADAM12-L* 3′UTR. Similar to miR-29, the miR-200 family is down-regulated in claudin-low tumors and cell lines. Thus, low expression levels of miR-200 family members, together with low expression of miR-29, may create permissive conditions for high expression of *ADAM12-L* in claudin-low tumors and cell lines.

To assess the clinical relevance of our results on the regulation of ADAM12-L expression in breast cancer cell lines, we analyzed publicly available expression data for a cohort of 100 breast cancer patients and found negative correlations between *ADAM12-L* mRNA and miR-29b, miR-200b, and miR-200c. Among these three miRNAs, miR-29b and miR-200c appear to be the most strongly correlated with *ADAM12-L* in breast tumors. Importantly, inhibition of endogenous miR-29b, and to a lesser extent miR-200c, in two different cell lines representing the basal subtype of breast cancer, SUM102PT and SUM149PT, led to increased expression of *ADAM12-L*. These findings support a role for the endogenous miR-29b and/or miR-200c in the regulation of *ADAM12-L* gene expression at the post-transcriptional level via targeting of the unique 3′UTRs of *ADAM12-L.* Since the translation product of *ADAM12-L* differs from the protein product of *ADAM12-S* in its biochemical properties, cellular localization, and most likely substrate specificity and function, better understanding of the mechanisms controlling expression of each splice variant is an important step in the research on ADAM12 in breast cancer.

## Conclusions

The *ADAM12-L* 3′UTR is a direct target of miR-29 and miR-200 family members. Since the miR-29 and miR-200 families play important roles in breast cancer progression, these results may help explain the different prognostic and chemopredictive values of *ADAM12-L* and *ADAM12-S* in breast cancer.

## Abbreviations

ADAM12: a disintegrin and metalloproteinase 12
miRNA: microRNA
3′UTR: 3′ untranslated region
EMT: epithelial-to-mesenchymal transition
ER: estrogen receptor
PR: progesterone receptor
TGFβ: transforming growth factor β
PyMT: Polyoma virus middle T antigen
SDS-PAGE: sodium dodecyl sulfate polyacrylamide gel electrophoresis
qRT-PCR: real-time quantitative reverse transcription polymerase chain reaction
FBS: fetal bovine serum
DPBS: Dulbecco’s phosphate buffered saline
DMEM: Dulbecco’s Modified Eagle Medium
AEBSF: 4-(2-Aminoethyl) benzenesulfonyl fluoride hydrochloride
HEPES: 2-(4-(2-hydroxyethyl)piperazin-1-yl)ethanesulfonic acid
Tris: 2-Amino-2-hydroxymethyl-propane-1,3-diol
